# Serum Biochemical Parameters of Broilers Affected by Wooden Breast Myopathy

**DOI:** 10.3390/ani14101499

**Published:** 2024-05-17

**Authors:** Eglė Lebednikaitė, Neringa Sutkevičienė, Toma Vilkonienė, Žana Balčiauskienė, Kęstutis Kučinskas, Lina Anskienė, Alius Pockevičius

**Affiliations:** 1 Department of Veterinary Pathobiology, Faculty of Veterinary Medicine, Lithuanian University of Health Sciences, Tilzes 18, 47181 Kaunas, Lithuania; alius.pockevicius@lsmu.lt; 2 Animal Reproduction Laboratory, Large Animal Clinic, Faculty of Veterinary Medicine, Lithuanian University of Health Sciences, Tilzes 18, 47181 Kaunas, Lithuania; 3 Vilnius Department of the State Food and Veterinary Service, Konstitucijos 23b, 08105 Vilnius, Lithuania; 4 Kaunas Department of the State Food and Veterinary Service, Veterinaru 14, Biruliskiu vil., Karmelavos p., 54469 Kaunas, Lithuania; 5 Department of Animal Breeding, Faculty of Animal Sciences, Lithuanian University of Health Sciences, Tilzes 18, 47181 Kaunas, Lithuania; lina.anskiene@lsmu.lt

**Keywords:** alanine aminotransferase, chicken, creatine kinase, wooden breast

## Abstract

**Simple Summary:**

Wooden breast myopathy is an abnormality that affects heavyweight, rapidly growing broilers’ breast muscle. The understanding of the exact cause of this muscle pathology remains incomplete, necessitating further investigation. Blood biochemical analysis is used to diagnose pathologies and understand disease processes. Therefore, the objective of this research was to determine and compare the changes in the blood serum biochemical parameters of broilers without myopathy and those affected by myopathy. Blood samples were collected from male and female 43-day-old broilers with an average live weight of 2.98–3.09 kg. The research results showed that birds with wooden breast had higher levels of creatine kinase, potassium, and alanine aminotransferase. Increased alanine aminotransferase indicated possible liver injury alongside wooden breast myopathy. Elevated creatine kinase and potassium suggested muscle damage, indicating CK as a potential biomarker for WB.

**Abstract:**

Wooden breast (WB) myopathy is a pathology of the *pectoralis major* muscle. Wooden breast is caused by multiple factors. The exact etiopathogenesis of this myodegenerative pathology is still unclear. Fast-growing commercial lines of broilers that are selected for high breast muscle yields are more susceptible to this myopathy. The biochemical analysis of blood is used to diagnose pathologies and understand disease processes. Therefore, the objective of this research was to determine and compare the changes in the blood serum biochemical parameters of Ross 308 chicken broilers without myopathy and those affected by WB myopathy. Blood samples were collected from male and female Ross 308 broilers that were 43 days old, with an average live weight of 2.98–3.09 kg. Representative blood samples were selected from broilers with WB (*n* = 33) and without WB (*n* = 33). In the laboratory, the blood was centrifugated, and biochemical tests were performed with an automated computerized biochemistry analyzer. The research results showed that broilers with WB had elevated blood serum levels of creatine kinase (CK) (*p* = 0.018), potassium (*p* = 0.010), and alanine aminotransferase (ALT) (*p* = 0.012). In conclusion, elevated serum levels of CK and potassium indicated that skeletal muscle cells were damaged. Moreover, increased ALT levels suggested a possible association between WB myopathy and liver damage. Additionally, these research findings underscore the diagnostic significance of CK and hint at its potential as a WB biomarker.

## 1. Introduction

Enormous improvements in the growth rates and muscle sizes of broiler chickens affect the poultry muscle structure, metabolism, and repair mechanisms and can cause myodegenerative pathologies—myopathies [1,2]. Wooden breast (WB) myopathy is detected by the visual examination and palpation of the breast muscle, the gold standard in slaughterhouses. Macroscopically, WB is characterized by pale and bulging hard areas of breast muscle with a surface hemorrhage and the presence of a light yellow, viscous exudate on the muscle’s surface [3]. It is known that WB lesions begin focally at approximately 2 weeks of age and then diffuse throughout the *pectoralis major* muscle during the bird’s life [4].

However, the exact aetiology of WB is still unknown [3]. Most research suggests that fast-growing commercial lines of broilers that are selected for high breast muscle yields and thicker fillets are more prone to this myopathy [4,5]. Researchers predict that genetics, localized muscle hypoxia, oxidative stress, circulatory deficiency, and impaired homeostasis are possible causes of WB in heavyweight broilers [6,7,8]. However, WB myopathy is not limited only to the *pectoralis major* muscle, and other organs may also play a role in the etiology of WB [9,10]. According to Lake et al. [10], when compared to that of unaffected birds, the lung histology of WB-affected birds showed greater foci of chondro-osseous metaplasia and sporadically localized multifocal lymphoplasmocytic phlebitis. Xing et al. (2021) [9] observed aberrant reactive oxygen species accumulation, the activation of antioxidant enzyme systems, and elevated content of malondialdehyde, lipid peroxidation, protein carbonyl, and 8-hydroxydeoxyguanosine in WB-affected birds. These findings suggest that liver damage occurs in WB-affected birds.

A histopathologic analysis of the *pectoralis major* muscle affected by WB has shown multifocal degeneration and necrosis, a loss of striation, and the infiltration of inflammatory cells, mainly macrophages and heterophils, within and around the degenerative fibers. According to the literature, rhabdomyolysis refers to the breakdown of the skeletal muscle tissue [3,11]. Various enzymes, muscle cell content, myoglobin, sarcoplasmic proteins, electrolytes, and various organic acids leak into the plasma or serum and may be indicative of injury to the muscles or other organs [11,12,13,14]. Increasing levels of alanine aminotransferase (ALT), aspartate aminotransferase (AST), creatine kinase (CK), and lactate dehydrogenase (LDH) are associated with liver or muscle damage. CK is a muscle-specific enzyme and is used as a diagnostic marker to differentiate between muscle and liver injuries [15].

The body’s osmotic pressure and acid–base balance are influenced by sodium, potassium, and calcium, which are found in tissues and cellular fluids. Potassium is responsible for the proper functioning of neural and muscle tissue and is involved in the muscle cell membrane potential (voltage gradient), the activation of numerous intracellular enzymes, and glucose and amino acid absorption and transport [16,17]. Phosphorus participates in the transmission of neural stimuli and is a constituent of cellular membranes and soft tissues. Magnesium activates a number of enzymes indispensable in carbohydrate and phosphorus–calcium metabolism, as well as providing an important function in the contraction process of muscles [18,19].

Overall, the biochemical analysis of animal blood is used for health assessment, the diagnosis of pathologies, and the understanding of disease processes [9]. However, limited research exists on the blood serum biochemical parameters associated with WB myopathy in broilers. Knowledge about the blood serum parameters of broilers with and without myopathy is crucial in determining the equilibrium status in the body, which reflects its metabolic activity. Therefore, the aim of this research was to determine and compare the changes in the blood serum biochemical parameters of Ross 308 chicken broilers with and without WB myopathy.

## 2. Materials and Methods

### 2.1. Ethical Considerations

The study did not require consent or ethical approval according to European Directive 2010/63/EU. All procedures involving animals were performed in strict accordance with the European slaughter regulations (CE n° 1099/2009 of 24 September 2009) for the protection of animals at the time of killing (Ref. Official Journal of the European Union L 303/1). Permission to obtain the samples was granted by the management of the slaughterhouses before the research commenced.

### 2.2. Broiler Husbandry and Blood Sample Collection

**Figure 1:**
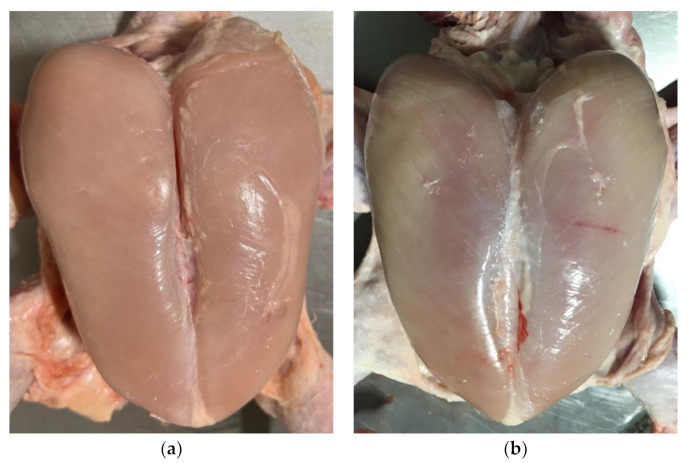
Macroscopic appearance of wooden breast. (**a**) *pectoralis major* muscle without wooden breast myopathy; (**b**) *pectoralis major* muscle affected by wooden breast myopathy. Source: the authors.

Chickens were bred and raised in a traditional intensive system in Lithuania, without antibiotics. The broilers were vaccinated against infectious bronchitis, infectious bursal disease, and Newcastle disease. The birds were 43 days old, with an average flock live weight of 2.98–3.09 kg. The broilers were slaughtered according to standard industrial practices and under the supervision of the official controlling veterinary authority. On a moving shackle line, the broilers were examined visually by a veterinarian, and 100 of them were selected randomly and tagged. After electrical stunning (150 mA, 400 Hz, 15–17 s, AC), 10 mL of blood was sampled directly during exsanguination via the carotid arteries and jugular veins into test tubes (treated with gel to help to separate the clot) (Venoject, Terumo Europe N. V., Leuven, Belgium) from each individual tagged bird. After death in the processing plant, all tagged birds were examined for WB. After the post-mortem examination of the *pectoralis major* muscle, representative blood samples (*n* = 66) were chosen and separated into two groups. The first group comprised those without WB myopathy (absence of WB) (*n* = 33): no pale areas, no hardness, and no thick liquid of the breast fillet (Figure 1a); the second group comprised those with WB myopathy (*n* = 33): hardness and pale muscle only in the cranial part of the breast fillet or throughout the breast fillet and a light yellow, viscous liquid on the breast surface (Figure 1b). No macroscopic pathologies were found in other organs in both groups.

### 2.3. Blood Serum Analysis

Samples of chicken blood were delivered to the laboratory in 2 h. The blood was centrifugated for 5 min at 3000 rotations per minute (rpm). Then, 1 mL of blood serum from each sample was separated and frozen at −20 °C for biochemical analysis. Biochemical blood tests were performed on all collected blood serum samples with an automated computerized biochemistry analyzer, the SELECTRA Junior (Netherlands, 2006), using Spinreact (Spain) reagents. The serum levels of urea, AST, ALT, alkaline phosphatase (ALP), iron, creatinine, calcium, magnesium, phosphorus, potassium, sodium, albumin, gamma-glutamyl transferase (GGT), high-density lipoprotein (HDL) cholesterol, triglycerides, total protein, and CK were determined. Additionally, the level of serum globulins was determined by subtracting the level of albumin from the total protein level.

### 2.4. Statistical Analysis

The blood serum biochemical parameters (*n* = 17) of each investigated chicken (*n* = 66) were collected in a database (Microsoft Excel 2021). In order to compute the data, the database was transferred and analyzed using SPSS 27.0 (SPSS Inc., Chicago, IL, USA). All measurements were tested for a normal distribution (Shapiro–Wilk test). The means and standard errors of the mean (M ± SEM) of the blood serum biochemical parameters were calculated. A two-tailed Student’s *t*-test was used to identify differences between the means of the investigated traits. The results were considered significant at *p* < 0.05.

## 3. Results

According to the study results, as seen in Table 1, the ALT concentration was 32.42% higher in the blood serum of broilers affected by WB compared to that in the serum of broilers without WB (*p* < 0.05). Additionally, 33.69% higher CK levels were observed in the blood serum of broilers affected by WB compared to those in the serum of broilers without WB (*p* < 0.05). However, AST, ALP, and GGT did not differ significantly between broilers affected and not affected by WB myopathy (*p* > 0.05).

The potassium levels were 27.36% higher in the blood serum of broilers affected by WB myopathy compared to that of broilers without myopathy (*p* ≤ 0.01). However, the iron, calcium, magnesium, phosphorus, and sodium concentrations in the serum did not differ significantly between broilers affected and not affected by WB myopathy (*p* > 0.05) (Table 2).

There were no significant differences (*p* > 0.05) observed in the concentrations of the investigated blood serum proteins synthesized in the liver between broilers affected by WB and those unaffected by it (Table 3).

## 4. Discussion

Rhabdomyolysis is the dissolution of the skeletal muscle, and, because of this, the muscle cell content, myoglobin, sarcoplasmic proteins, and electrolytes leak into the extracellular fluid and circulation [11]. The most common cause of rhabdomyolysis is direct traumatic injury. However, this condition can also be the result of drugs, toxins, infections, muscle ischemia, electrolyte and metabolic disorders, genetic disorders, and temperature-induced states such as malignant hyperthermia [20]. Adenosine triphosphate (ATP) depletion due to muscle cell damage causes sarcolemma disruption. Therefore, fluids are drawn into the cell, along with sodium. This process causes the swelling of the cells, disturbs the integrity of the cell membrane (sarcolemma), and disrupts the ion channels. Leukocytes migrate into the damaged muscle due to reperfusion. This migration of inflammatory cells increases the levels of cytokines, prostaglandins, and free radicals. Moreover, elevated amounts of these substances cause further muscle fiber necrosis and release muscle breakdown products into the bloodstream. Potassium, myoglobin, phosphate, organic acids, and various enzymes, such as CK and LDH, leak into the plasma or serum [12,14]. Furthermore, CK is a muscle-specific enzyme used to diagnose muscle injury [15]. Additionally, WB-affected breast muscle is histologically characterized by increased degenerative and atrophic muscle fibers, vacuolar degeneration and necrosis, hyalinization, lipidosis, fibrosis, and the infiltration of macrophages and heterophils [3,21,22]. In this study, the concentration of CK was significantly higher in the blood serum of broilers affected by WB compared to that in the serum of broilers without WB. Therefore, the findings of this study justify that rhabdomyolysis occurs in wooden breast myopathy. Moreover, this finding indicates the potential utility of CK as a biomarker for WB. Several authors have published similar results. Kawasaki et al. [23] found significantly higher CK values in a group of 20-day-old WB-affected birds (42,360 IU/L) than in those unaffected by WB (10,164 IU/L). Kawasaki et al. [23] and Meloche et al. [24] suggest that the blood plasma CK increases significantly with the increasing age of unaffected birds and WB and white striping (WS) scores. In addition, according to Kong et al. [13], CK could be a candidate blood marker for the prediction of breast muscle defects in 42-day-old broilers and assist in genetic selection in broiler breeding. Generally, identifying such biomarkers has a great deal of potential to help poultry specialists with earlier decision-making regarding the slaughter of affected animals, thereby reducing the production costs. Additionally, the early identification of WB-associated biomarkers could inform adjustments in management practices during the growth phase, thus enhancing the overall quality of the meat and possibly slowing down the myopathy formation process.

Furthermore, it is important to note that the release of enzymes into the bloodstream is not exclusive to muscle damage but also occurs due to liver tissue injury, especially with aminotransferases such as AST and ALT [25]. According to the literature, both aminotransferases are highly concentrated in the liver [26]. In this study, ALT was significantly higher in the blood serum of broilers affected by WB. However, AST was elevated in the blood serum of broilers affected by WB but not statistically different compared to that in the serum of broilers without WB. There are some differences between those two enzymes. AST is represented in the heart, skeletal muscle, kidneys, brain, and red blood cells, while ALT has low concentrations in the skeletal muscle and kidneys. ALT is more specific to liver damage [26,27]. The liver is a multifunctional organ. It is responsible for most of the synthesis, metabolism, excretion, and detoxification processes in the body. The liver plays an important role in digestion and metabolism, regulating the production, storage, and release of carbohydrates, lipids, and proteins [28]. Researchers have linked WB myopathy to broilers’ homeostasis dysregulation and liver damage [29]. According to the research results, it may be suggested that liver failure also occurs in fast-growing broilers affected by WB myopathy. However, not only pathological causes but also several physiological factors, such as sex, age, body mass index, and extreme physical exertion, could influence the ALT levels in the blood serum, especially in mammalian studies [30,31]. Therefore, in future studies, it is crucial to explore additional physiological factors, such as nutrition, environmental stressors, and genetic factors, that could also contribute to fluctuations in ALT levels, in order to enhance the interpretation of elevated serum ALT levels in chickens affected by WB.

Additionally, significantly higher potassium levels were found in the blood serum of broilers exhibiting signs of WB. Similar results were found by Livingston et al. [32] and Lake J.A. [10]. Livingston et al. [32] found a level of potassium in 42-day-old broilers without WB of 4.75 mmol/L, while, in those affected by mild, moderate, or severe WB, it was significantly higher—6.25 mmol/L, 5.76 mmol/L, and 5.87 mmol/L, respectively. Lake et al. [10] also found significantly different potassium levels: 5.04 mmol/L in broilers affected by WB and 4.86 mmol/L in unaffected broilers. Furthermore, according to Lake et al. [10], chickens with WB exhibit not only higher levels of potassium but also higher pCO2 and a lower pH. Lake et al. [10] reported higher concentrations of potassium and pCO2 along with a lower pH, suggesting insufficient respiratory gas exchange. One limitation of this study, however, is that only the biochemical blood profile was analyzed, which restricted the analysis to the mineral levels, without considering other potential indicators of altered oxygen metabolism or the acid–base balance.

The concentrations of urea, albumin, globulin, total protein, HDL cholesterol, triglycerides, and creatinine in the blood serum were not different between broilers affected and unaffected by WB. These results are similar to the findings of Kuttappan et al. [33] and Amaral et al. [34]. According to Kuttappan et al. [33], there were no differences in the levels of urea, albumin, triglycerides, and creatinine of broilers with normal and severe degrees of white striping. Amaral et al. [34] compared the biochemical serum parameters of broilers with and without dorsal cranial myopathy. The albumin, total protein, and HDL cholesterol levels were not significantly different between broilers with and without dorsal cranial myopathy. Urea and creatinine levels are helpful indicators of renal function. They show the rate of glomerular filtration, as well as the balance between urea generation, urea excretion, and protein catabolism [35]. However, no significant changes in the indicators of renal function were observed.

## 5. Conclusions

In conclusion, the results confirm that the CK levels were elevated in the blood serum of broilers affected by WB myopathy. The potassium and ALT concentrations were also higher in the blood samples that were collected from broilers with WB. The elevated serum CK indicated that the skeletal muscle cells were damaged and therefore potassium had leaked into the extracellular space. Additionally, the increased levels of ALT in the blood serum of broilers affected by WB suggest a possible association between WB myopathy and liver damage. Moreover, the elevated CK concentration in the blood of affected birds suggests the potential usefulness of CK as a biomarker for WB. It may be an important tool for poultry specialists in the detection of myopathy that might help to reduce the production costs.

## Figures and Tables

**Table 1:** Means and standard errors of the mean (M ± SEM) of the blood serum enzymes of broilers without myopathy (control group) and those affected by wooden breast myopathy (WB group).

Enzymes U/L	Control Group (*n* = 33)	WB Group (*n* = 33)	*p*-Value
Alanine aminotransferase (ALT)	25.45 ± 2.255	33.70 ± 2.267	0.012
Aspartate aminotransferase (AST)	620.92 ± 24.026	622.09 ± 17.415	0.969
Creatine kinase (CK)	63,725.21 ± 5212.258	85,196.42 ± 7119.934	0.018
Alkaline phosphatase (ALP)	6679.06 ± 586.131	8636.70 ± 917.477	0.077
Gamma-glutamyl transferase (GGT)	27.06 ± 1.027	25.15 ± 1.045	0.197

**Table 2:** Means and standard errors of the mean (M ± SEM) of the blood serum minerals of broilers without myopathy (control group) and those affected by wooden breast myopathy (WB group).

Mineral	Control Group (*n* = 33)	WB Group (*n* = 33)	*p*-Value
Iron, µg/dL	87.64 ± 1.846	84.01 ± 2.597	0.260
Calcium, mmol/L	2.31 ± 0.059	2.31 ± 0.045	0.987
Magnesium, mmol/L	1.10 ± 0.018	1.14 ± 0.019	0.123
Phosphorus, mmol/L	2.69 ± 0.041	2.75 ± 0.045	0.345
Potassium, mmol/L	37.68 ± 3.024	47.99 ± 2.451	0.010
Sodium, mmol/L	144.70 ± 1.864	145.64 ± 1.783	0.717

**Table 3:** Means and standard errors of the mean (M ± SEM) of the blood serum proteins, high-density lipoprotein (HDL) cholesterol, triglycerides, and creatinine of broilers without myopathy (control group) and those affected by wooden breast myopathy (WB group).

Attribute	Control Group (*n* = 33)	WB Group (*n* = 33)	*p*-Value
Urea, mmol/L	1.35 ± 0.037	1.36 ± 0.031	0.755
Albumin, g/L	16.75 ± 0.232	17.08 ± 0.225	0.311
Globulin, g/L	16.10 ± 0.468	16.05 ± 0.381	0.936
Total protein, g/L	32.84 ± 0.639	33.12 ± 0.516	0.732
High-density lipoprotein (HDL) cholesterol, mmol/L	1.70 ± 0.051	1.72 ± 0.045	0.745
Triglycerides, mg/dL	66.52 ± 3.335	68.45 ± 4.994	0.748
Creatinine, µmol/L	31.12 ± 0.396	31.06 ± 0.454	0.920

## Data Availability

Data are contained within the article.

## Abbreviations

WB	wooden breast
CK	creatine kinase
ALT	alanine aminotransferase
AST	aspartate aminotransferase
LDH	lactate dehydrogenase
AC	alternating current
rpm	rotations per minute
ALP	alkaline phosphatase
GGT	gamma-glutamyl transferase
HDL	high-density lipoprotein
M	mean
SEM	standard error of the mean
ATP	adenosine triphosphate
WS	white striping
pCO2	partial pressure of carbon dioxide

## References

[1] MacRae V.E., Mahon M., Gilpin S., Sandercock D.A., Mitchell M.A. (2006). Skeletal muscle fibre growth and growth associated myopathy in the domestic chicken (*Gallus domesticus*). Br. Poult. Sci..

[2] Sandercock D.A., Barker Z.E., Mitchell M.A., Hocking P.M. (2009). Changes in muscle cell cation regulation and meat quality traits are associated with genetic selection for high body weight and meat yield in broiler chickens. Genet. Sel. Evol..

[3] Sihvo H.K., Immonen K., Puolanne E. (2014). Myodegeneration with fibrosis and regeneration in the pectoralis major muscle of broilers. Vet. Pathol..

[4] Huang X., Ahn D.U. (2018). The incidence of muscle abnormalities in broiler breast meat—A review. Korean J. Food Sci. Anim. Resour..

[5] Havenstein G.B., Ferket P.R., Qureshi M.A. (2003). Carcass composition and yield of 1957 versus 2001 broilers when fed representative 1957 and 2001 broiler diets. Poult. Sci..

[6] Mutryn M.F., Brannick E.M., Fu F., Lee W.R., Abasht B. (2015). Characterization of a novel chicken muscle disorder through differential gene expression and pathway analysis using RNA-sequencing. BMC Genom..

[7] Clark D.L., Velleman S.G. (2016). Spatial influence on breast muscle morphological structure, myofiber size, and gene expression associated with the wooden breast myopathy in broilers. Poult. Sci..

[8] Malila Y., Thanatsang K., Arayamethakorn S., Uengwetwanit T., Srimarut Y., Petracci M., Strasburg G.M., Rungrassamee W., Visessanguan W. (2019). Absolute expressions of hypoxia inducible factor-1 alpha (HIF1A) transcript and the associated genes in chicken skeletal muscle with white striping and wooden breast myopathies. PLoS ONE.

[9] Xing T., Pan X., Zhang L., Gao F. (2021). Hepatic Oxidative Stress, Apoptosis, and Inflammation in Broiler Chickens With Wooden Breast Myopathy. Front. Physiol..

[10] Lake J.A., Brannick E.M., Papah M.B., Lousenberg C., Velleman S.G., Abasht B. (2020). Blood Gas Disturbances and Disproportionate Body Weight Distribution in Broilers With Wooden Breast. Front. Physiol..

[11] Stanley M., Chippa V., Aeddula N.R., Quintanilla Rodriguez B.S., Adigun R. (2023). Rhabdomyolysis. StatPearls.

[12] Hoffman W.E., Solter P.F., Kaneko J.J., Harvey J.W., Bruss M.L. (2008). Diagnostic enzymology of domestic animals. Clinical Biochemistry of Domestic Animals.

[13] Kong F., Zhao G., He Z., Sun J., Wang X., Liu D., Zhu D., Liu R., Wen J. (2021). Serum Creatine Kinase as a Biomarker to Predict Wooden Breast in vivo for Chicken Breeding. Front. Physiol..

[14] Huerta-Alardín A.L., Varon J., Marik P.E. (2005). Bench-to-bedside review: Rhabdomyolysis—An overview for clinicians. Crit. Care.

[15] Lumeij J.T., Kaneko J.J., Harvey J.W., Bruss M.L. (1997). Avian clinical biochemistry. Clinical Biochemistry of Domestic Animals.

[16] Leeson S., Summers J.D. (2001). Minerals. Scott‘s Nutrition of the Chicken.

[17] Oliveira J., Albino L., Rostagno H., Páez L., Carvalho D. (2005). Dietary levels of potassium for broiler chickens. Braz. J. Poult. Sci..

[18] Siegel H.S. (1995). Stress, strains and resistance. Br. Poult. Sci..

[19] Bláhová J., Dobšíková R., Strakova E., Suchý P. (2007). Effect of Low Environmental Temperature on Performance and Blood System in Broiler Chickens (*Gallus domesticus*). Acta Vet. Brno.

[20] Cervellin G., Comelli I., Lippi G. (2010). Rhabdomyolysis: Historical background, clinical, diagnostic and therapeutic features. Clin. Chem. Lab. Med..

[21] Velleman S.G., Clark D.L. (2015). Histopathologic and myogenic gene expression changes associated with wooden breast in broiler breast muscles. Avian Dis..

[22] Soglia F., Mudalal S., Babini E., Di Nunzio M., Mazzoni M., Sirri F., Cavani C., Petracci M. (2016). Histology, composition, and quality traits of chicken Pectoralis major muscle affected by wooden breast abnormality. Poult. Sci..

[23] Kawasaki T., Iwasaki T., Yamada M., Yoshida T., Watanabe T. (2018). Rapid growth rate results in remarkably hardened breast in broilers during the middle stage of rearing: A biochemical and histopathological study. PLoS ONE.

[24] Meloche K.J., Fancher B.I., Emmerson D.A., Bilgili S.F., Dozier W.A. (2018). Effects of reduced dietary energy and amino acid density on Pectoralis major myopathies in broiler chickens at 36 and 49 days of age1. Poult. Sci..

[25] Li S., Muhammad I., Yu H., Sun X., Zhang X. (2019). Detection of Aflatoxin adducts as potential markers and the role of curcumin in alleviating AFB1-induced liver damage in chickens. Ecotoxicol. Environ. Saf..

[26] Giannini E.G., Testa R., Savarino V. (2005). Liver enzyme alteration: A guide for clinicians. Can. Med. Assoc. J..

[27] Wroblewski F. (1958). The clinical significance of alterations in transaminase activities of serum and other body fluids. Adv. Clin. Chem..

[28] Zaefarian F., Abdollahi M.R., Cowieson A., Ravindran V. (2019). Avian Liver: The Forgotten Organ. Animals.

[29] Abasht B., Mutryn M.F., Michalek R.D., Lee W.R. (2016). Oxidative stress and metabolic perturbations in wooden breast disorder in chickens. PLoS ONE.

[30] Moriles K.E., Azer S.A. (2024). Alanine Amino Transferase. StatPearls.

[31] Bussler S., Vogel M., Pietzner D., Harms K., Buzek T., Penke M., Händel N., Körner A., Baumann U., Kiess W. (2018). New pediatric percentiles of liver enzyme serum levels (alanine aminotransferase, aspartate aminotransferase, γ-glutamyltransferase): Effects of age, sex, body mass index, and pubertal stage. Hepatology.

[32] Livingston M.L., Landon C.D., Barnes H.J., Brake J., Livingston K.A. (2019). Dietary potassium and available phosphorous on broiler growth performance, carcass characteristics, and wooden breast. Poult. Sci..

[33] Kuttappan V.A., Huff G.R., Huff W.E., Hargis B.M., Apple J.K., Coon C., Owens C.M. (2013). Comparison of hematologic and serologic profiles of broiler birds with normal and severe degrees of white striping in breast fillets. Poult. Sci..

[34] Amaral P.C., Zimermann C., Santos L.R., Noro M., Prá M.D., Pilotto F., Rodrigues L.B., Dickel E.L. (2017). Evaluation of physiological parameters of broilers with dorsal cranial myopathy. Braz. J. Poult. Sci..

[35] Scholz M.C. (2005). Laboratory tests defined. PCRI.

